# DBR1 siRNA inhibition of HIV-1 replication

**DOI:** 10.1186/1742-4690-2-63

**Published:** 2005-10-18

**Authors:** Ying Ye, Jessica De Leon, Noriko Yokoyama, Yathi Naidu, David Camerini

**Affiliations:** 1 Department of Molecular Biology & Biochemistry, 2230 McGaugh Hall, University of California, Irvine, Irvine, CA 92697-3900, USA

## Abstract

**Background:**

HIV-1 and all retroviruses are related to retroelements of simpler organisms such as the yeast Ty elements. Recent work has suggested that the yeast retroelement Ty1 replicates via an unexpected RNA lariat intermediate in cDNA synthesis. The putative genomic RNA lariat intermediate is formed by a 2'-5' phosphodiester bond, like that found in pre-mRNA intron lariats and it facilitates the minus-strand template switch during cDNA synthesis. We hypothesized that HIV-1 might also form a genomic RNA lariat and therefore that siRNA-mediated inhibition of expression of the human RNA lariat de-branching enzyme (DBR1) expression would specifically inhibit HIV-1 replication.

**Results:**

We designed three short interfering RNA (siRNA) molecules targeting DBR1, which were capable of reducing DBR1 mRNA expression by 80% and did not significantly affect cell viability. We assessed HIV-1 replication in the presence of DBR1 siRNA and found that DBR1 knockdown led to decreases in viral cDNA and protein production. These effects could be reversed by cotransfection of a DBR1 cDNA indicating that the inhibition of HIV-1 replication was a specific effect of DBR1 underexpression.

**Conclusion:**

These data suggest that DBR1 function may be needed to debranch a putative HIV-1 genomic RNA lariat prior to completion of reverse transcription.

## Background

Retrotransposons resemble retroviruses and contain long terminal repeats (LTR). They are mobile DNA elements that replicate via RNA intermediates. The Ty1 retroelement is among the best characterized of the retrotransposons of the yeast *Saccharomyces cerevisiae *[1-3]. Using a genetic screen aimed at identifying cellular factors involved in Ty1 transposition, Chapman and Boeke found that S. cerevisiae DBR1 mutants had lower Ty1 transposition frequency and were viable [4]. Dbr1 protein is a 2'-5' phosphodiesterase that cleaves intron RNA lariat branch points after splicing, facilitating ribonucleotide turnover [4,5]. DBR1 mutants produce mature mRNA, but accumulate intron lariats [3,4]. Mutations in DBR1 inhibit both Ty1 transposition and cDNA formation. Cheng and Menees provided evidence that Ty1 transcripts contain a 2'-5' branch characteristic of an RNA lariat, and demonstrated that the genomic RNA lariat is an unanticipated intermediate in the life cycle of Ty1 [2]. The location of this branch suggests that it may play a role during the formation of Ty1 cDNA by facilitating the transfer of nascent minus-strand of Ty1 cDNA from the upstream region of the Ty1 RNA template to the downstream region [2,3]. The similarity of Ty1 to animal retroviruses further suggests that the branch may be widely conserved among retroviruses as well as retroelements.

Human immunodeficiency virus (HIV) reverse transcription of the RNA genome into DNA is performed by the viral enzyme reverse transcriptase (RT). The primer for reverse transcription is a cellular tRNA. Retroviruses, long terminal repeat (LTR) retrotransposons, and long interspersed nucleotide element retrotransposons use cellular tRNAs to initiate cDNA synthesis [6-8]. Different tRNAs are used by different retroviruses and retrotransposons [9]. Early in the viral life cycle, the tRNA primes minus-strand strong-stop DNA synthesis, whereby the 5' end of the viral positive sense RNA genome is copied into minus-strand cDNA by RT, while the RNA template is degraded by the RNAse-H activity of RT. After minus-strand strong-stop DNA synthesis, a template shift from the 5' LTR to the 3' LTR is required to continue synthesis of the complete minus-strand cDNA, a requirement for converting the viral RNA genome into the proviral DNA genome. This "template switch" is required to synthesize the complete DNA genome, but its precise mechanism has never been identified. The recent work of Menees and colleagues showed that the 5' nucleotide (nt) of Ty1 RNA forms a 2'-5' bond with a nt at the beginning of R region in the 3' LTR of the same RNA, creating a lariat. The properties of the lariat suggest it forms by a novel mechanism and that branching and debranching may play roles in Ty1 reverse transcription at the minus-strand transfer step.

RNAi is a mechanism of gene silencing that has been widely used to study gene function *in vitro *[10-14]. RNAi occurs in cells via complex endogenous machinery that recognizes double stranded RNA, cleaves it into small fragments (19–21 nucleotides), and then uses those fragments as guides to specifically degrade RNA species displaying complementary sequence. The small fragments, called short interfering RNA (siRNA), can be introduced directly into mammalian cells to enter this pathway to induce the specific degradation of intracellular RNA. SiRNA-induced RNAi mediated degradation of DBR1 mRNA was used to study the role of DBR1 protein in HIV-1 replication.

## Results

### Transfection of GHOST cells with DBR1 siRNAs resulted in suppression of DBR1 mRNA

Three DBR1 siRNAs, targeted to DBR1 sequence positions 215 to 235, 436 to 456 and 971 to 991 were designed and inserted into pHyper (analogous to pSuper, obtained from Dr. V. Planelles of the University of Utah). For each DBR1 siRNA expression plasmid, two 64 nucleotide (nt) oligonucleotides were annealed to create 5' Bgl-II and 3' Hind III sites flanking a 21 nt target sequence in the DBR1 gene, a central 9 nt loop followed by a 21 nt antisense copy of the target sequence. The annealed 64 mers were cloned between the Bgl-II and Hind-III sites of the pHyper vector, as previously described for pSuper [15]. These three DBR1 siRNA plasmids were designated pHyper-D1, pHyper-D2 and pHyper-D3.

**Figure 1:**
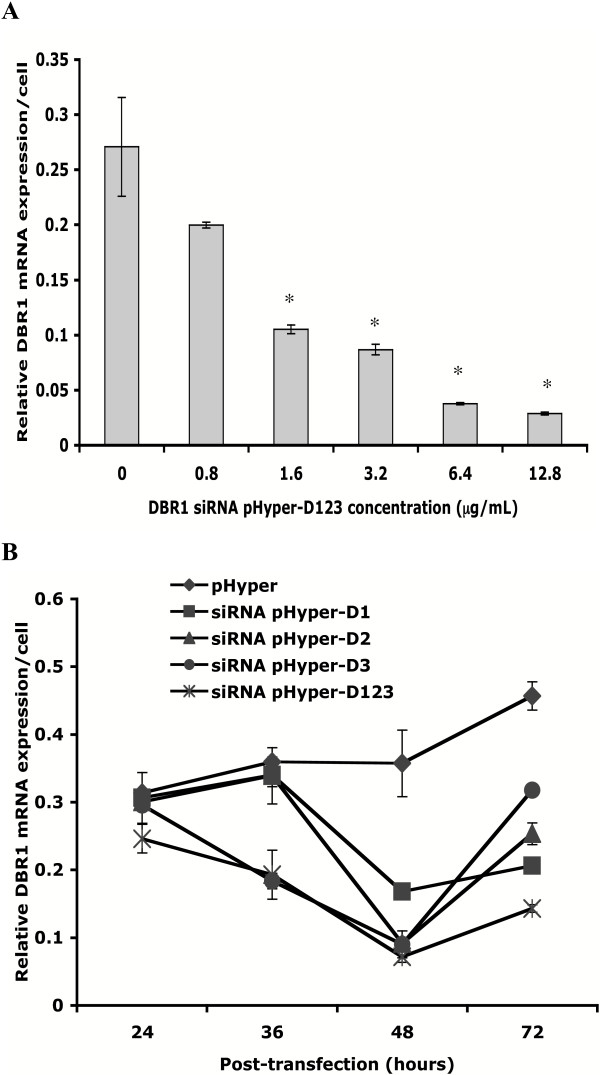
**Suppression of *DBR1 *mRNA expression by DBR1 siRNA**. **(A) **GHOST-R5X4 cells were transfected with various concentrations of DBR1 siRNA pHyper-D123 (0.8 μg/ml, 1.6 μg/ml, 3.2 μg/ml, 6.4 μg/ml, 12.8 μg/ml) or mock transfected. Forty-eight hours post transfection, RNA was isolated, treated with Dnase I and used as template in real-time quantitative RT-PCR. Each reaction included 300 ng RNA, 0.5 μM each gene-specific primer for DBR1 and GAPDH amplification. GAPDH was used as an internal control. **(B) **GHOST-R5X4 cells were transfected with 12.8 μg/ml DBR1 siRNA pHyper-D1, pHyper-D2, pHyper-D3, pHyper-D123 or control pHyper and cells were collected at the indicated time points. RNA was isolated, treated with Dnase I and analyzed by real-time quantitative RT-PCR. Results shown are from triplicate samples in a representative experiment. Error bars indicate standard deviations. Results that are significant by Student's t-test are indicated by asterisks (p < 0.05).

GHOST-R5X4 cells were transfected with various amounts of all three DBR1 siRNA expression plasmids combined in equal proportions (pHyper-D123) or control vector pHyper for forty-eight hours with Lipofectamine. To evaluate the effects of DBR1 siRNA, total RNA was extracted and real-time quantitative RT-PCR was performed with DBR1 specific primers. The DBR1 mRNA level was downregulated to a maximum of 80% following transfection with 12.8 μg/ml pHyper-D123 (Fig 1A). Next we examined the kinetics of DBR1 mRNA knockdown in GHOST-R5X4 cells transfected with each DBR1 siRNA plasmid separately (pHyper-D1, pHyper-D2, pHyper-D3) or together (pHyper-D123) compared to cells transfected with the control vector pHyper. We analyzed DBR1 message levels at various time points post-transfection by real-time quantitative RT-PCR. The data revealed that transfection of cells with any of the three DBR1 siRNA expression plasmids resulted in a reduction in the amount of DBR1 mRNA compared to cells transfected with the control vector pHyper (Fig 1B). This effect was maximal forty-eight hours post-transfection. Moreover, we found that the mixture of all three DBR1 siRNA expressing plasmids was the most effective and reduced the DBR1 mRNA level by more than 80%.

### Knockdown of DBR1 or over expression of DBR1 is not toxic to cells

**Figure 2:**
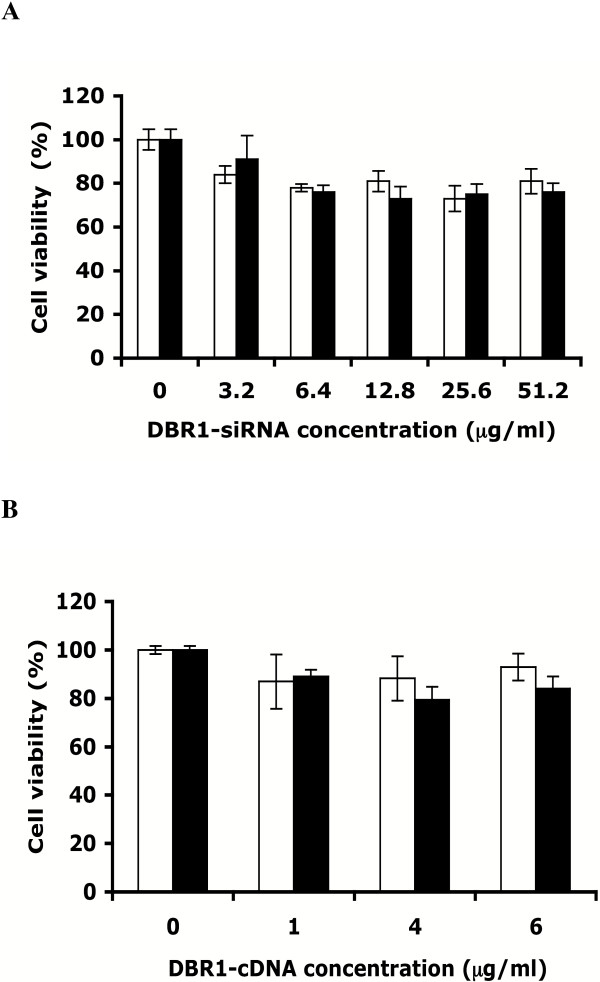
**Determination of cell viability**. **(A) **GHOST-R5X4 cells were transfected with 3.2 μg/ml, 6.4 μg/ml, 12.8 μg/ml, 25.6 μg/ml or 51.2 μg/ml DBR1 siRNA pHyper-D123 (black bars) or the same concentrations of pHyper alone (white bars) as a control. **(B) **GHOST-R5X4 cells were transfected with 1 μg/ml, 4 μg/ml, 6 μg/ml CDM9-DBR1 (black bars) or the same concentrations of CDM9 alone (white bars) as a control. After forty-eight hours cellular viability was assayed with a commercial MTS assay kit (Promega). Results shown are from triplicate samples in a representative experiment. Error bars indicate standard deviations.

We used a commercial assay of cellular mitochondrial metabolism to determine whether DBR1 siRNA or DBR1 cDNA expression had adverse or cytotoxic effects. GHOST-R5X4 cells were transfected with pHyper-D123 and pHyper as a control or CDM9-DBR1 and CDM9 as a control. Forty-eight hours later, we assayed mitochondrial electron transport by its ability to reduce the tetrazolium salt, MTS, to produce formazan using a commercial kit. We did not detect significant cytotoxic effects following the addition of 3.2, 6.4, 12.8, 25.6, or 51.2 μg/ml of DBR1 siRNA pHyper-D123 and 1, 4, or 6 μg/ml of CDM9-DBR1 compared to the same concentration of each control plasmid (Fig 2).

### DBR1 knockdown inhibits HIV-1 replication in GHOST cells

**Figure 3:**
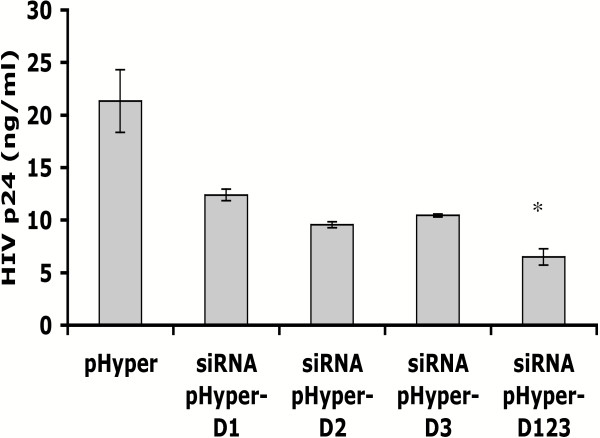
**DBR1 siRNA inhibition of HIV-1 replication**. GHOST-R5X4 cells were transfected with 12.8 μg/ml DBR1 siRNA pHyper-D1, pHyper-D2, pHyper-D3, pHyper-D123 or control pHyper. Forty-eight hours later, cells were infected with HIV-1 (JR-CSF) for twenty-four hours and p24 in supernatants was analyzed using a commercial HIV-1 p24 ELISA kit. All ELISA measurements were done in triplicate. Error bars indicate +/- standard deviation. Results marked with an asterisk are significant by Student's t-test (p < 0.05).

To test whether DBR1 siRNA was capable of inhibiting HIV-1 replication, GHOST-R5X4 cells were transfected with pHyper-D1, pHyper-D2, pHyper-D3, pHyper-D123 or pHyper alone for forty-eight hours, then infected with R5-HIV-1 (JR-CSF). Twenty-four hours after HIV-1 infection, cell supernatants were collected to detect extracellular HIV-1 by ELISA for the viral capsid protein, p24. These data showed more than a 70% decrease in HIV-1 replication in GHOST-R5X4 cells expressing three DBR1 siRNA molecules compared to vector transfected cells (Fig 3). We also measured HIV-1 p24 in cell supernatants that were collected two and three days post-infection. Less suppression of viral replication was observed at these times, however, probably because the levels of DBR1 mRNA had returned to near normal (Fig. 1B and data not shown).

### DBR1 over-expression counteracts the effect of DBR1 siRNA on HIV-1 replication

**Figure 4:**
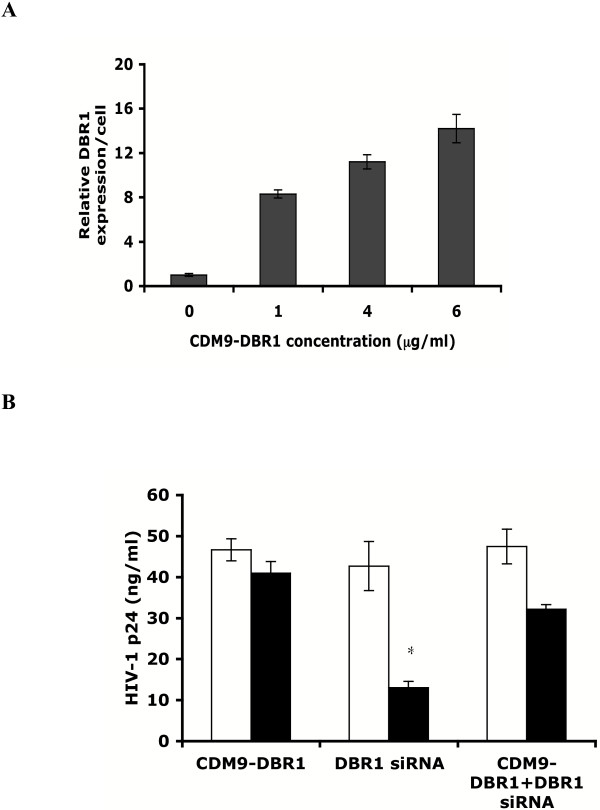
***DBR1 *over-expression does not affect HIV-1 replication**. **(A) **GHOST-R5X4 cells were transfected with various amounts of CDM9-DBR1 (1 μg/ml, 4 μg/ml, 6 μg/ml) or mock transfected for forty-eight hours. Total RNA was isolated and DBR1 expression was quantified by real-time RT-PCR. **(B) **GHOST-R5X4 cells were transfected with CDM9-DBR1 (black bar, CDM9-DBR1), control vector CDM9 (white bar, CDM9-DBR1) or DBR1 siRNA pHyper-D123 (black bar, DBR1 siRNA), control vector pHyper (white bar, DBR1 siRNA), or cotransfected CDM9-DBR1 and DBR1 siRNA pHyper-D123 (black bar, CDM9-DBR1 + DBR1 siRNA), control vector CDM9 and pHyper (white bar, CDM9-DBR1 + DBR1 siRNA) for forty-eight hours and then infected with HIV-1 (JR-CSF) for twenty-four hours. Supernatants were collected and p24 was assayed using a commercially available HIV-1 p24 ELISA kit. All ELISA measurements were done in triplicate. Error bars indicate standard deviations. The asterisk denotes p < 0.05 by Student's t-test.

We next queried whether DBR1 over-expression would affect HIV-1 replication alone or when cotransfected with DBR1 siRNA. GHOST-R5X4 cells were transfected with a human DBR1 cDNA expressed by the plasmid vector, CDM9 at three concentrations or with vector alone. RNA was extracted forty-eight hours later and quantified by real-time quantitative RT-PCR with DBR1 specific primers (Fig. 4A). We observed a dose dependent increase in DBR1 mRNA expression as expected. Subsequently, GHOST-R5X4 cells were transfected with CDM9-DBR1, control vector CDM9, or DBR1 siRNA pHyper-D123, control vector pHyper or cotransfected with CDM9-DBR1 and DBR1 siRNA pHyper-D123, control vector CDM9 and pHyper, for forty-eight hours and then infected with HIV-1 (JR-CSF). To evaluate HIV-1 replication, the supernatants were analyzed twenty-four hours post infection for the presence of viral capsid, p24, with a commercial ELISA kit (Fig. 4B). We did not observe a significant effect of DBR1 overexpression on HIV-1 replication in GHOST-R5X4 cells. In contrast, the three DBR1 siRNA's significantly repressed HIV-1 replication and this inhibition could be reversed by cotransfection of DBR1 cDNA. This indicates that the DBR1 siRNA inhibited HIV-1 replication specifically, by lowering DBR1 expression.

### DBR1 siRNA suppresses HIV-1 replication during reverse transcription

**Figure 5:**
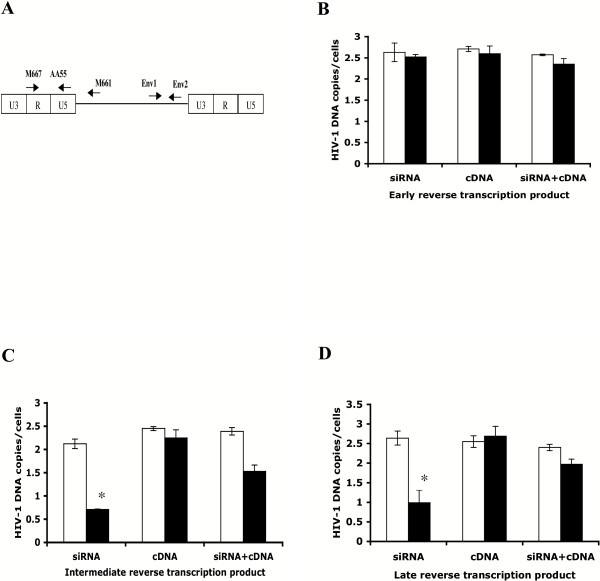
**DBR1 siRNA suppresses HIV-1 reverse transcription after minus-strand strong-stop DNA formation**. **(A) **Location of primers. The oligonucleotide primers M667/AA55 specific for the R/U5 region of the LTR were used to detect early reverse transcripts (strong-stop DNA) **(B) **and *env *primers Env1/Env2 to were used detect intermediate reverse transcripts **(C)**. LTR/*gag *primers M667/M661 were chosen to detect complete first-strand viral cDNA **(D)**. In **B**, **C **and **D**, GHOST-R5X4 cells were transfected with DBR1 siRNA pHyper-D123 (black bars, siRNA), control vector pHyper (white bars, siRNA) or CDM9-DBR1 (black bars, cDNA), control vector CDM9 (white bars, cDNA) or co-transfected with CDM9-DBR1 and DBR1 siRNA pHyper-D123 (black bars, siRNA + cDNA), control vectors CDM9 and pHyper (white bars, siRNA + cDNA) for forty-eight hours, followed by infection with Dnase-treated HIV-1 (JR-CSF). Twenty-four hours later, the cells were lysed and DNA was isolated for real time quantitative PCR to evaluate the synthesis of HIV-1 cDNA. Copies of HIV-1 DNA were normalized against copies of β-globin. Error bars indicate standard deviations. Asterisks denote p < 0.05 by Student's t-test.

To more precisely determine the stage at which HIV-1 replication is inhibited by degradation of DBR1 mRNA, we transfected GHOST-R5X4 cells with DBR1 siRNA pHyper-D123 or control vector pHyper for forty-eight hours, then infected the cells with HIV-1, and harvested them twenty-four hours post-infection. The cells were lysed and DNA was isolated to evaluate the synthesis of HIV-1 cDNA by real-time quantitative PCR. The oligonucleotide primers M667 and AA55 specific for the R and U5 regions of the HIV-1 LTR respectively were used to detect early reverse transcription products, also known as strong-stop DNA, *env *primers Env1 and Env2 were used to detect intermediate reverse transcription products, while the LTR-R region and *gag *primers M667 and M661 were chosen to detect completely synthesized viral cDNA (Fig. 5A). Copies of HIV-1 were normalized against copies of β-globin to control for differences in cell number between samples. The results showed that early, products of reverse transcription were differentially affected by expression of DBR1 siRNA compared to intermediate and late reverse transcription products. The accumulation of HIV-1 strong-stop DNA was not affected by DBR1 siRNA expression (Fig. 5B), while the level of intermediate length and complete HIV-1 cDNA molecules was decreased similarly in the presence of DBR1 siRNA twenty-four hours post-infection (Fig. 5C,5D). This difference implies that the lariat debranching activity of DBR1 is needed after strong-stop DNA synthesis but prior to reverse transcription of the *env *gene or the completion of reverse transcription. To assay the specificity of inhibition of HIV-1 reverse transcription by DBR1 siRNA, we transfected GHOST-R5X4 cells with CDM9-DBR1, control vector CDM9 or with DBR1 siRNA pHyper-D123, control vector pHyper or cotransfected with CDM9-DBR1 and DBR1 siRNA pHyper-D123 or control vector CDM9 and pHyper for forty-eight hours and then infected with HIV-1 (JR-CSF). As shown in Fig 5, DBR1 over-expression did not significantly affect HIV-1 reverse transcription. Moreover, the effect of DBR1 siRNA expression on HIV-1 cDNA synthesis could be reversed by cotransfection of a DBR1 cDNA. This result indicates that the inhibition of HIV-1 reverse transcription by DBR1 siRNA was mediated specifically, by suppression of DBR1 expression.

## Discussion

SiRNAs are widely used for targeting and silencing genes by RNA interference. The recent discovery that exogenously delivered siRNA can trigger RNAi in mammalian cells allows the use of this technology in research and perhaps eventually as a therapeutic tool. Inhibition of HIV-1 replication with RNAi has already been demonstrated with siRNAs against a variety of structural or regulatory genes including *gag*, *pol*, *nef*, *vif*, *tat*, *env *and *rev *[16-22]. These results demonstrate that siRNA directed to an HIV-1-specific gene could inhibit viral replication. In addition to targeting viral genes, many studies also investigated the efficacy of siRNAs in down regulating host cell molecules necessary for HIV-1 infection [23-28]. An advantage in targeting the interaction of cellular molecules with HIV-1 is that broad-spectrum efficacy against all clades of HIV-1 might be more readily achievable and the frequency of escape mutants might be lower. In this report, we have shown that siRNAs targeted to the host cellular gene DBR1 specifically decreased the amount of HIV-1 cDNA and capsid protein produced.

It has been previously found that the yeast gene DBR1 affects Ty1 transposition [1,4,29]. This gene encodes the RNA debranching enzyme, which hydrolyzes the 2'-5' bonds of intron RNA lariat branch points. A recent report suggested that the debranching enzyme influences Ty1 transposition because the Ty1 genomic RNA forms a lariat intermediate during cDNA synthesis [2]. This lariat intermediate was proposed to be formed by a 2'-5' phosphodiester bond between the 5' end of the genome and the first nucleotide in the 3' R region. Since retroviruses like HIV-1 and retroelements of *Saccharomyces cerevisiae *like Ty1 replicate via similar mechanisms and have similar LTR structures, we tested whether reduction in human DBR1 affected HIV-1 replication. We used three DBR1 siRNAs to specifically degrade DBR1 mRNA and measured the effects of DBR1 down regulation on HIV-1 replication.

We demonstrated that DBR1 siRNA expression reduced the level of DBR1 mRNA in a dose and time dependent manner and decreased the amount of virus produced from infected cells. This DBR1 siRNA-mediated inhibition of HIV-1 replication occurred after strong-stop DNA formation but prior to reverse transcription of the *env *gene or the completion of minus-strand synthesis. We found that DBR1 siRNA inhibited the formation of *env *gene cDNA and complete HIV-1 cDNA twenty-four hours post-infection (Fig. 5). In contrast, there was little effect on the synthesis of minus-strand strong-stop DNA. To determine if the lack of virus production and inhibition of reverse transcription were specifically due to DBR1 siRNA, we transfected DBR1 cDNA alone or with DBR1 siRNA and analyzed HIV replication. DBR1 over-expression alone had little effect on HIV-1 replication but it blocked the inhibitory effect of DBR1 siRNA when cotransfected. These results demonstrate the specific inhibition of HIV-1 reverse transcription by DBR1 siRNA after strong-stop DNA formation – perhaps during the minus-strand template switch. Nevertheless, we cannot exclude suppression of viral replication at other stages after strong-stop DNA formation.

In summary, our studies show that siRNA targeted to the human DBR1 gene specifically inhibited HIV-1 replication during reverse transcription. Although we do not know the role of the putative HIV-1 genomic RNA lariat in viral replication, it is likely that further investigation with DBR1 siRNA will provide insights into the mechanism of HIV-1 cDNA synthesis.

## Conclusion

We have demonstrated that suppression of the host RNA lariat de-branching enzyme (DBR1) with siRNA specifically inhibits HIV-1 replication. Further studies showed DBR1 siRNA suppresses HIV-1 replication during reverse transcription. A recent report found that the minus-strand template switch in the yeast retroelement Ty1 is accomplished via an RNA lariat intermediate, which is formed by a 2'-5' phosphodiester bond between the 5' end and 3' LTR of the genomic RNA. HIV-1 is similar to retroelements like Ty1and our results suggest that HIV-1 may utilize a similar RNA lariat intermediate during minus-strand transfer. This finding may have profound implications for the development of new therapeutics for AIDS.

## Methods

### Construction of siRNA expression vectors

The mammalian expression vector, pHyper (analogous to pSuper) was obtained from Dr. Vicente Planelles of the University of Utah and used for expression of siRNA. For each siRNA expression plasmid, a pair of 64 nucleotide oligonucleotides were annealed to create a 5' Bgl-II and 3' Hind III site flanking a 21 nt target sequence in the DBR1 gene, a central 9 nt loop followed by a 21 nt antisense copy of the target sequence. Three pairs of cDNA oligonucleotides, targeting the human DBR1 gene at different locations were synthesized by MWG Biotech (Irvine, CA). Each pair of oligonucleotides was annealed at 90°C for 4 minutes, then at 70°C for 10 minutes, cooled to 37°C, and incubated for 20 minutes. The annealed dsDNA oligonucleotides were ligated into the pHyper vector between the Bgl-II and Hind III sites. These three 21 nt DBR1 sequences, *D1*, AACGAGGCGGATCTACGCTGC; *D2*, AAGGATCGGTGGAATCTCTGG; *D3*, AATGTGACTGGGCGCCTGTGG, were used to create three DBR1-siRNA plasmids, designated pHyper-D1, D2 and D3.

### Cell culture, DNA transfection, viral stock preparation and infection

GHOST-R5X4 cells were cultured in Iscove's modified Dulbecco's medium (IMDM) containing 10% fetal bovine serum (FBS), 50 μg/ml gentamicin and 500 μg/ml of G418. Human embryonic kidney 293T cells were cultured in Dulbecco's modified Eagle medium (DMEM) supplemented with 10% FBS and 50 μg/ml gentamicin. GHOST-R5X4 cells were plated at 95% confluency for transfection and transfected using Lipofectamine (Invitrogen) according to the manufacturer's instructions. Forty-eight hours after transfection, cells were collected. Viral stocks were prepared by transfecting 293T cells (seeded at 9 × 10^6 ^cells per T75 flask) with 100 μg of πSV-JR-CSF plasmid coprecipitated with calcium phosphate. Two days post-transfection, culture supernatant was collected and frozen at -80°C until needed. HIV-1 virions in the supernatant were quantified using HIV-1 p24 ELISA kit (Perkin Elmer).

### Cell viability assay

Cell viability assays were conducted with CellTiter 96 aqueous cell proliferation assay kit (Promega) according to the manufacturer's specifications. Briefly, cells were plated into flat-bottom 96-well plates at a density of 2 × 10^3 ^cells/well and allowed to attach overnight. Cells were then transfected with different concentrations of pHyper-DBR1 siRNA and pHyper as a control or CDM9-DBR1 and CDM9 as a control. After forty-eight hours of incubation, 20 μL of MTS reagent was added to each well, the plate was incubated for three hours at 37°C. A 490 nm absorbance value was determined using a Model-550 ELISA plate reader (Beckman Instruments Inc.). The percentage of viable cells was calculated as: (*Abs*_sample _- *Abs*_blank_)/(*Abs*_control _- *Abs*_blank_) × 100.

### RNA isolation and real-time quantitative RT-PCR

Total RNA was isolated using Trizol reagent (Life Technologies) according to the manufacturer's instructions. The amount of extracted RNA was quantified by measuring the absorbance at 260 nm. One microgram of RNA was treated with 1 unit Dnase I (Invitrogen) in a volume of 10 μl to remove contaminating DNA (37°C for 15 min, 75°C for 5 min). Three hundred ng of Dnase I treated RNA was reverse transcribed using a two-step reverse transcription kit (Applied Biosystems) in a final volume of 10 μl. Reverse transcription was performed for 60 min at 37°C. The total cDNA volume of 10 μl was frozen until real-time quantitative PCR was performed. After thawing for PCR experiments, the cDNA was diluted in 90 μl of distilled water and 2 μl of diluted cDNA was used for each PCR reaction. Real-time quantitative PCR was performed using ABI Prism 7700 sequence detection system, (PE Applied Biosystems) for amplification and detection. PCR conditions were as follows: initial denaturation at 95°C for 10 min then 40 rounds of cycling at 95°C for 15 sec and 60°C for 60 sec. Each PCR reaction in triplicate contained 15 μl SYBR green PCR master mix (Applied Biosystems), and 0.3 μM of each gene-specific primer for human DBR1 and glyceraldehyde-3-phosphate-dehydrogenase (GAPDH) in a 30 μl reaction volume. Forward and reverse primer sequences for amplifying DBR1 were GGAAACCATGAAGCCTCAAA (nt 247–266) and CCGATCCTTACACCTCGGTA (nts 444–425), respectively. Forward and reverse primer sequences for amplifying GAPDH were GGTGGTCTCCTCTGACTTCAA (nt 840–860) and GTTGCTGTAGCCAAATTCGTTGT (nt 966–944). A normalized DBR1 value was calculated by dividing the DBR1 copy number (determined from the appropriate standard curve) by the GAPDH (endogenous reference) copy number.

### P24 ELISA

HIV-1 p24 ELISA was performed using a commercially available kit (Perkin Elmer) according to the manufacturer's instructions. For measuring p24 in the supernatants, 10^3 ^fold dilutions of the supernatants were used. All ELISA measurements were done in triplicate.

### Quantitative real-time PCR

At each time point, 3.0 × 10^6 ^cells were lysed in 100 μl of 100 μg/ml proteinase K in 10 mM Tris-HCl, pH 8.0 at 56°C for 1 hour, followed by heat inactivation at 95°C for 10 min. Each PCR reaction contained 15 μl SYBR green PCR master mix (Applied Biosystems), 5 μl of cell lysate, 0.3 μM of each primer in a 30 μl reaction volume. HIV-1 specific primers AA55, CTGCTAGAGATTTTCCACACTGAC (nts 635–612) and M667, GGCTAACTAGGGAACCCACTG (nts 496–516) were used to detect minus strand strong stop. Env1, GGCAGGGATACTCACCCTTATCG (nts 8337–8360) and Env2, GGATTTCCCACCCCCTGCGTCCC (nts 8590–8566) were used to detect intermediate lengthe reverse transcription products. M661, CCTGCGTCGAGAGAGAGCTCCTCTGG (nts 695–672) and M667 were used to detect complete synthesis of first-strand cDNA. Copies of HIV-1 DNA were normalized against copies of β-globin using primers LA1 globin, ACACAACTGTGTTCACTAGC and LA2 globin, CAACTTCATCCACGTTCACC directed to β-globin [30].

### DBR1 cDNA isolation and expression

A 1.7 kb cDNA fragment encoding human DBR1 was amplified by PCR from a human PHA-stimulated T cell cDNA library [31]. Amplification reactions were performed in a 25 μl mixture containing 0.625 Units FailSafe PCR Enzyme Mix (Epicentre), 500 nM forward (GAATTCGCCACCATGCGGGTGGCTGTGGCTGGCTGCTGCCACGG) and reverse (AACAAGTAAATCATCTTAAGCTGCATCG) primers in FailSafe PCR Premix-E buffer. The amplification reactions were carried out with the following program: one cycle of 2 min at 92°C, 35 cycles consisting of 30 sec at 95°C, 1 min at 55°C, 2 min at 72°C and a final extension step of 10 min at 72°C. PCR products were separated on 1% agarose gel pre-stained with ethidium bromide. A gel cleanup kit (Eppendorf) was used to purify and elute the PCR product DNA fragment for cloning and sequencing. The fragment was inserted between the Xho1 and Not1 sites of CDM9 and the sequence was found to be identical to the DBR1 sequence in the NCBI database. CDM9 is a derivative of CDM8 that contains a larger region of the human CMV immediate early region including the first intron and non-coding exon [32]. Various amounts of CDM9-DBR1 (1, 4 and 6 μg/ml) were used to transfect GHOST-R5X4 cells with Lipofectamine (Invitrogen) according to manufacturer's instructions, CDM9 alone was used as a control. Total cellular RNA was extracted and real-time quantitative RT-PCR was performed to quantify DBR1 expression.

## Competing interests

The author(s) declare that they have no competing interests.

## Authors' contributions

YY carried out most of the experiments and drafted this manuscript. JDL performed most of the real-time PCR and analyzed the data. NY isolated the DBR1 cDNA and constructed some of the siRNA expression vectors. YN designed the DBR1 siRNAs and helped construct the siRNA expression vectors. DC conceived of the study, participated in its design and coordination and helped to revise the manuscript. All living authors read and approved the final manuscript.
